# Enhancing the Power Output of InSe-Based Screen-Printed Flexible Thermoelectric Generators through a Bi–Te–Co-Doping Strategy

**DOI:** 10.1021/acsomega.5c09692

**Published:** 2025-12-15

**Authors:** Manasa R. Shankar, Ashwatha Narayana Prabhu, Ramakrishna Nayak

**Affiliations:** † Department of Physics, 125853 Manipal Institute of Technology, Manipal Academy of Higher Education, Manipal 576104, India; ‡ Department of Humanities and Management, Manipal Institute of Technology, Manipal Academy of Higher Education, Manipal, Karnataka 576104, India

## Abstract

The advancement of flexible thermoelectric generators (FTEGs) is hindered by the brittleness, rigidity, and complex processing of conventional materials, as well as challenges in achieving both mechanical durability and efficient charge transport. Although single-element doping, alloying, and nanostructuring have been explored to enhance thermoelectric performance, they often require complex synthesis or cause trade-offs between electrical and thermal transport. Here, we show that Bi/Te codoping in indium selenide (InSe) provides a more effective approach by simultaneously optimizing carrier concentration and introducing phonon scattering centers, thereby achieving balanced improvements in the Seebeck coefficient, electrical conductivity, and thermal conductivity. Bi/Te codoped InSe powders were synthesized via a conventional solid-state reaction method, and flexible FTEGs were subsequently fabricated using a facile and scalable screen-printing technique, providing a cost-effective and industrially viable alternative. Structural analysis confirms the formation of phase-pure hexagonal InSe, with enhanced crystallinity achieved at an optimal 4% Bi doping level. Hall effect measurements reveal that codoping significantly improves electrical properties, resulting in a high Seebeck coefficient (-452 μV/K), increased voltage output (47 mV), and superior power output (∼0.14 nW at Δ*T* = 116 °C) for the In_0.96_Bi_0.04_Se_0.97_Te_0.03_ composition, representing a 6-fold increase in power output compared to pristine InSe. Moreover, the fabricated devices exhibit exceptional flexibility and mechanical reliability, maintaining electrical performance with ∼5% resistance variation under bending and 500 mechanical cycles. This work not only demonstrates a high-performing n-type InSe-based flexible thermoelectric material but also establishes a practical route toward scalable, wearable energy-harvesting devices.

## Graphical abstract

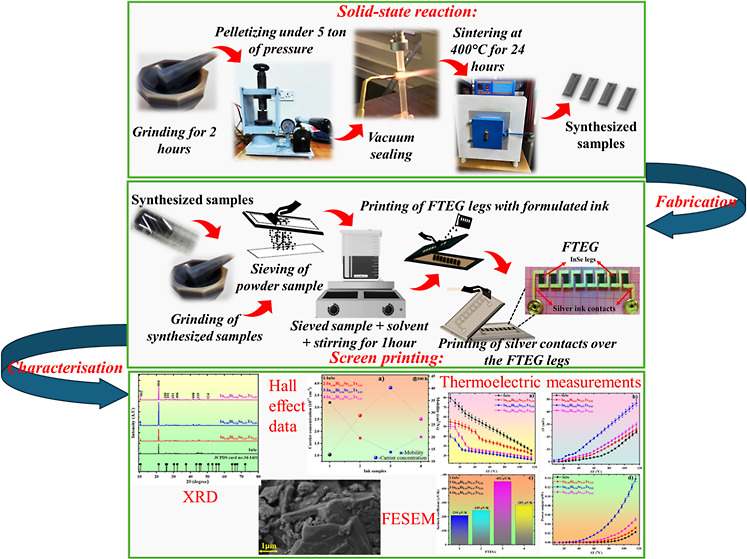

## 1 Introduction

The ever-growing demand for self-powered and maintenance-free energy systems in wearable electronics, implantable biomedical devices, smart textiles, environmental sensors, and wireless communication nodes has spurred extensive research into energy harvesting technologies.^1^ Among various strategies, such as piezoelectric, triboelectric, and pyroelectric mechanisms, thermoelectric generators (TEGs) stand out due to their ability to directly convert temperature gradients into electrical energy via the Seebeck effect.^2^ The human body alone generates up to ∼100 W of thermal energy during routine activities, and harvesting even a small fraction of this heat can be sufficient to power miniature electronic devices, thus eliminating the need for frequent battery replacement or recharging.^3^ This makes FTEGs promising for wearable and skin-integrated electronics. The output performance of FTEGs is primarily governed by the Seebeck coefficient (S), electrical conductivity (σ), and thermal conductivity (κ), as a high S and σ combined with a low κ enable larger voltage generation and higher power output. Therefore, optimizing these parameters is essential to realize efficient flexible devices for practical low-grade heat harvesting.

Significant advances have been made in the development of thermoelectric (TE) materials, especially chalcogenide-based semiconductors such as Bi_2_Te_3_, PbTe, and Sb_2_Te_3_, which exhibit excellent thermoelectric properties in bulk and thin-film forms. Despite their high thermoelectric performance, they suffer from rigidity, toxicity, and fabrication challenges, limiting their integration into flexible or wearable platforms. Organic thermoelectric materials, on the other hand, offer mechanical flexibility and easy processing but generally exhibit poor thermoelectric efficiency.^2^ To overcome these limitations, research has recently focused on developing fully inorganic thermoelectric systems that combine flexibility with high electrical performance and environmental stability. Notably, Ag_2_Se-based thin films have demonstrated remarkable potential for near-room-temperature thermoelectric applications, achieving a power density exceeding 65 W m^–2^ and maintaining 90% of their performance after 1000 bending cycles, owing to optimized film orientation and controlled Ag interstitial content.^4^ Similarly, Bi_2_Te_3_ and BiSbTe-based flexible thin films have achieved a figure of merit (ZT) as high as 1.11 at 393 K with excellent bending stability (<5% resistance change after 1000 cycles) and power densities over 1038 μW cm^–2^ when integrated with n-type Ag_2_Se films.^5,6^ These recent advancements highlight the growing potential of high-performance inorganic thin films for flexible and wearable thermoelectric devices, such as self-powered sensors and IoT applications.

Indium selenide (InSe), a III–VI layered semiconductor,^7^ is an attractive candidate in this context due to its low thermal conductivity (∼0.37–1.2 W m^1–^ K^–1^),^8^ moderate bandgap (∼1.3 eV),^9^ and high carrier mobility. Its layered structure with weak van der Waals interlayer bonding enables mechanical flexibility, while its ambient stability and visible-range direct bandgap make it viable for real-world applications.^10^ Although InSe’s moderate bandgap leads to a low intrinsic carrier concentration (∼10^14^ cm^–3^),^11^ this drawback can be addressed by doping and codoping strategies to tune its electrical transport behavior. Previous reports have demonstrated that both cationic (e.g., Bi)^12^ and anionic (e.g., Te, S)^13^ doping in InSe effectively reduces lattice thermal conductivity and enhances power factors through point defect scattering and improved carrier transport, including our earlier work on bulk samples has demonstrated that codoping strategies, such as incorporating Bi and Te, can enhance electrical transport properties and suppress lattice thermal conductivity through enhanced phonon scattering.^14^

Although several studies have explored the thermoelectric optimization of InSe-based systems, most efforts have been limited to rigid bulk materials, with minimal progress in translating these advances into flexible device architectures. The mechanical integrity, electrical stability, and interfacial compatibility of InSe under bending conditions remain insufficiently studied. Moreover, no prior reports exist on flexible thermoelectric generators (FTEGs) derived from screen-printed InSe or Bi/Te codoped InSe materials. Addressing these gaps requires a scalable and cost-effective fabrication approach capable of producing uniform, adherent, and mechanically stable thermoelectric films suitable for flexible device integration.

This work presents the prior demonstration of fully inorganic FTEGs fabricated using screen-printed InSe and Bi/Te codoped InSe materials. Two key strategies distinguish this study. First, the use of the screen printing method offers a simple, low-cost, and scalable route to fabricate large-area FTEGs under ambient conditions.^15^ Unlike conventional high-temperature and high-pressure synthesis techniques such as spark plasma sintering or thermal evaporation, screen printing enables room-temperature processing on flexible substrates, making it suitable for mass production and integration into wearable systems.^16^ Second, codoping InSe with Bi and Te allows fine-tuning of electrical and thermal transport properties.^14^ By combining the advantages of screen-printed processing and Bi/Te codoping in a purely inorganic system, this study demonstrates a promising pathway for the development of scalable, flexible, and efficient thermoelectric generators. The results highlight the potential of codoped InSe-based FTEGs as viable candidates for next-generation wearable and portable energy harvesting applications.

## 2 Materials Used and Experimental Procedure

### 2.1 Materials

High-purity elemental precursors, tellurium (Te, 99.999%), selenium (Se, 99.999%), bismuth (Bi, 99.999%), and indium (In, 99.9%), were procured from Thermo Fisher Scientific (USA) and employed without further refinement for the synthesis of thermoelectric materials. The screen-printable ink formulation incorporated diacetone alcohol (DAA) as the volatile solvent and cellulose acetate propionate (CAP) as the rheology modifier and binding agent, both sourced from Sigma-Aldrich. Device fabrication was carried out on a flexible, optically transparent polyethene terephthalate (PET) substrate with a nominal thickness of 100 μm, supplied by Venlon Polyester Film Ltd., India. For the formation of conductive interconnects and stable electrical contacts, a commercially available silver-based conductive ink (Loctite ECI 1010 E&C, Henkel, India) was employed.

### 2.2 Synthesis Methodology and Characterization of Pristine and Bi/Te Co-Doped InSe

Polycrystalline samples of undoped InSe and Bi/Te codoped compositions with the general formula In_1–*x*_Bi_*x*_Se_0.97_Te_0.03_ (*x* = 0, 0.02, 0.04, 0.06) were synthesized via a conventional solid-state reaction route, following the methodology described in our previous work.^14^ This approach ensures phase purity and effective substitutional incorporation of dopant atoms within the InSe crystal lattice. Bi^3+^ ions were selected to partially substitute In^3+^ cations, while Te^2–^ ions replace Se^2–^ anions, exploiting comparable ionic radii and valence states to favor site-specific incorporation and minimal lattice strain.^14^ The required quantities of high-purity elemental precursors, In, Bi, Se, and Te, were procured from commercial sources, as detailed in Section 2.1. Each composition was synthesized in a 5 g batch, and the precise stoichiometric calculations were based on atomic weight percentages, as presented in Table 1.

**Table 1:** Composition of InSe, Bi/Te Co-Doped InSe Inks

ink name	In (g)	Se (g)	Bi (g)	Te (g)	ink vehicle (g)
InSe	2.962	2.037			2
In_0.98_Bi_0.02_Se_0.97_Te_0.03_	2.854	1.942	0.106	0.097	2
In_0.96_Bi_0.04_Se_0.97_Te_0.03_	2.769	1.924	0.210	0.096	2
In_0.94_Bi_0.06_Se_0.97_Te_0.03_	2.686	1.906	0.312	0.095	2

The elemental powders were weighed accurately and ground in an agate mortar for approximately 2 h to promote uniform distribution. The homogenized powder was then compacted into pellets using a hydraulic press under an applied pressure of 5 tons. The pressed pellets were evacuated in quartz ampules (∼10^–4^ Torr) to mitigate oxidation and suppress volatilisation of selenium and tellurium during the sintering process. The sealed ampules were subjected to an initial sintering cycle at 400 °C for 24 h, followed by natural furnace cooling. After sintering, the pellets were ground for an additional 1 h to enhance microstructural homogeneity and promote complete phase formation. A second pelletization was followed by a sintering at 400 °C for 12 h to ensure densification of the samples. The crystalline phase and structural uniformity of the synthesized InSe samples were previously validated using X-ray diffraction (XRD), revealing a hexagonal phase without the presence of any secondary phases. Since the diffraction profiles obtained in the current investigation closely replicate those earlier reported in,^14^ they are not reiterated here.

### 2.3 Formulation and Characterization of Screen-Printable Inks for Pristine and Bi/Te Co-Doped InSe

High-purity bulk InSe and Bi/Te codoped InSe samples were synthesized through a conventional solid-state reaction route. The resulting pellets were mechanically ground and passed through a fine mesh sieve to achieve a uniform particle size distribution suitable for ink formulation. The screen-printable inks were prepared by dissolving 15 wt % CAP in 85 wt % DAA, forming a stable organic binder-solvent matrix. Table 1 details the specific compositions of the formulated inks corresponding to the Bi/Te codoped In_1–*x*_Bi_*x*_Se_0.97_Te_0.03_ system with *x* = 0, 0.02, 0.04, and 0.06. The sieved InSe-based powders were thoroughly blended with the CAP + DAA ink vehicle using magnetic stirring for 1 h to achieve uniform particle dispersion and stable colloidal suspension. Ink viscosity was precisely tuned to lie within the optimal range of 1750–2000 cP,^17^ enabling reliable transfer and film uniformity during screen printing.

Structural analysis of the inks was carried out via X-ray diffraction (XRD, Rigaku Ultima IV) over a 2θ range of 10°–90° with a scanning increment of 0.02°/min to confirm crystallographic phase integrity. Surface morphology and microstructural attributes of the inks were investigated using field-emission scanning electron microscopy (FESEM) (Carl Zeiss Sigma), facilitating insight into the surface morphology of the films.

### 2.4 Procedure for Screen Printing

FTEGs were fabricated via a tailored screen-printing process. The prepared thermoelectric ink was systematically applied onto a mesh screen embedded with a defined geometric pattern. Using a rubber squeegee, uniform pressure was exerted to facilitate the transfer of the ink through the open areas of the mesh onto a PET substrate, strictly adhering to the stencil design. Following deposition, the printed films were left to dry at ambient temperature to facilitate solvent evaporation and stabilize the ink film. Subsequently, thermal post-treatment was performed in a hot-air oven at 60 °C for 1 h to enhance film adhesion and eliminate residual solvents. The curing temperature was carefully selected to prevent thermal distortion of the PET substrate, which begins to deform at temperatures exceeding ∼130 °C. This screen-printing methodology was adapted and refined based on our prior experience in the fabrication of printable thermoelectric devices.^18,19^

### 2.5 Development of Printed Thermoelectric Layers for Hall Effect and FTEG Fabrication

To examine the electrical transport characteristics, Hall measurement samples were prepared using the optimized printable inks. High-resolution screen meshes (120/cm, Shebro, India) and stencils were digitally designed using Inkscape software with square patterns of 10 mm × 10 mm. Both indirect and direct patterning techniques were employed to ensure precise and reproducible stencil formation for uniform ink deposition. Screen printing was carried out on flexible PET substrates employing a flat-edge rubber squeegee (75 Shore A hardness), with 12 successive print passes to attain uniform film thickness. Postprinting, the films were thermally dried and their thickness quantified using a precision Mitutoyo 547–301 thickness gauge. Hall effect measurements were conducted at ambient temperature using the van der Pauw method, employing a Keithley 6220 current source under a magnetic field of 0.6 T and a current of 50 mA to extract parameters such as charge carrier density and mobility.

The fabrication of FTEGs was centered around the development of functional thermoelectric inks. Each FTEG unit consisted of eight thermoelectric elements, each measuring 3 mm × 10 mm and printed in 12 successive layers onto a 100 μm-thick PET substrate, as illustrated in Figure 1. Commercially available silver paste was used to print four-layer interconnects to ensure low-resistance electrical pathways. All printed FTEGs maintained standardized dimensions of 15 mm × 55 mm, allowing for consistent performance comparison across devices.

**Figure 1:**
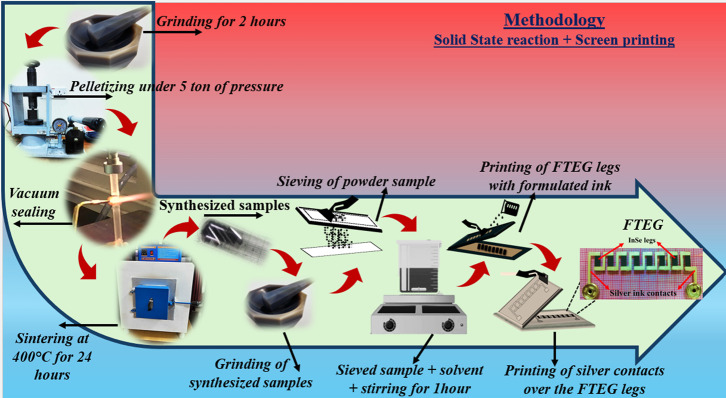
Overview of the fabrication workflow for InSe-based flexible thermoelectric generators, starting from material synthesis to ink formulation and screen printing of FTEG.

### 2.6 Characterization of FTEG

A previously developed experimental setup^18^ was employed to measure the Seebeck coefficient and output power of the screen-printed FTEGs using the formulated inks. The measurement system included two K-type digital thermocouples (Lutron TM-902C) for monitoring the temperature difference across the device and a Keithley 2001 source meter for capturing the open-circuit voltage and internal resistance with high precision. A thermal gradient was established by heating one side of the FTEG on a hot plate, while the opposite side was maintained at room temperature. Voltage and resistance values were recorded under steady-state conditions following established protocols reported in the literature.^17,19–23^ To examine the mechanical robustness of the printed devices, flexibility tests were conducted using a custom-built bending apparatus introduced in our earlier work.^18^ The FTEGs were subjected to systematic single-cycle bending at incremental cycle counts (0, 100, 200, 300, 400, and 500 cycles) with a bending radius of approximately 1.5 cm. Electrical measurements were taken during bending using a Fluke 179 digital multimeter to assess both structural stability and the retention of electrical performance under repeated mechanical strain. All measurements were repeated three times to ensure reproducibility, and the observed variation in results was within 10%, confirming the reliability and consistency of the data. Figure 1 presents a step-by-step depiction of the methodology and workflow implemented in this work.

## 3 Results and Discussion

### 3.1 Structural Analysis via X-ray Diffraction (XRD)

Following the ink formulation, the solvent was evaporated by drying the ink mixture under ambient conditions. The resulting solid residues were then manually ground to obtain fine powders suitable for XRD analysis. The structural characteristics of the resulting powders were examined using XRD, and the corresponding diffraction patterns are illustrated in Figure 2a. All observed peaks correspond well with the standard hexagonal phase of indium selenide (space group: *P*6_3_/*mmc*, JCPDS no. 34-1431), confirming phase purity without any detectable secondary phases. A prominent diffraction peak at 21.3°, associated with the (004) lattice plane, serves as a distinct marker of layered Se–In–In–Se stacking structure intrinsic to InSe crystals.^24^ Interestingly, with increasing Bi concentration, a distinct nonmonotonic trend is evident in the (004) peak intensity. Specifically, the peak intensity increases up to 4% Bi doping, followed by a slight reduction at higher doping levels. This behavior suggests that moderate Bi incorporation promotes preferential grain orientation and improved crystallinity along the (004) plane, likely due to lattice relaxation and enhanced grain coalescence during ink drying and annealing. However, at higher doping concentrations, excessive lattice strain introduced by dopant oversaturation disrupts the long-range ordering, resulting in diminished peak intensity. This improvement suggests that at moderate doping levels, Bi atoms are uniformly substituted into the InSe lattice, leading to reduced lattice imperfections and promoting grain growth. However, a decline in peak intensity at higher doping concentrations (6%) implies the onset of lattice strain and defect formation, which may hinder further enhancement in crystallinity.^25^

**Figure 2:**
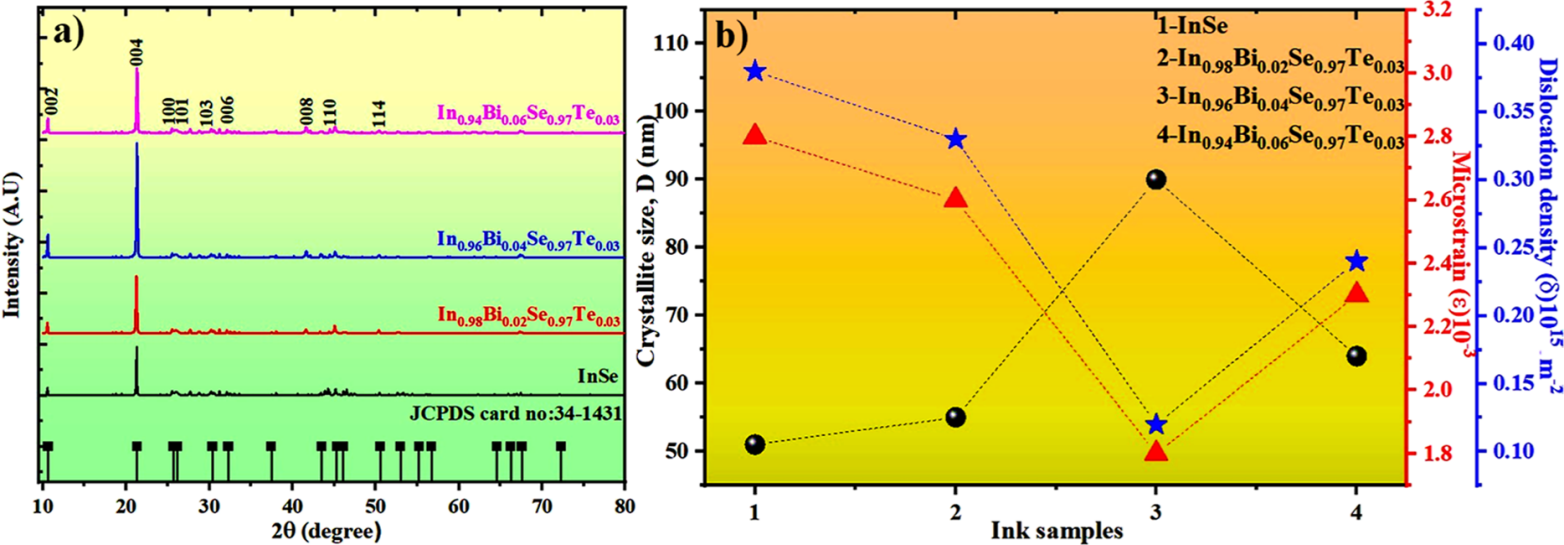
(a) XRD peak pattern, (b) plot of D Vs δ and ε of InSe and Bi/Te codoped InSe ink samples.

The variations observed in the XRD peak intensities of Bi/Te codoped InSe (Figure 2b) reflect intrinsic microstructural modifications induced by dopant incorporation. To gain deeper insight into these structural changes, key parameters such as average crystallite size, lattice strain, and dislocation density were quantified for the formulated ink samples. The crystallite size (D) estimation via the Scherrer equation is expressed in eq 1 ^26^

1 $$D=\frac{0.9\lambda }{\beta \cos\theta }$$

This calculation considers the X-ray wavelength (λ), the full width at half-maximum (β) of the diffraction peaks, and the Bragg diffraction angle (θ) expressed in radians. Additionally, dislocation density (δ), is determined using eq 2 ^26^

2 $$\delta =\frac{1}{D^{2}}$$

Similarly, the average microstrain (ε) is given by eq 3 ^26^

3 $$\varepsilon =\frac{\beta }{4\tan\theta }$$

The crystallite sizes of the ink samples were found to range from 51 to 90 nm. Notably, the sample with 4% Bi doping exhibited the largest crystallite size (90 nm), as evidenced by the narrowest and highest intensity XRD peak (004), indicating pronounced grain growth and superior crystalline ordering at this composition. Correspondingly, this sample also demonstrated the lowest dislocation density (0.12 × 10^15^ m^–2^) among all the doped variants, suggesting a substantial reduction in structural defects and dislocation networks.^25^ The observed decrease in defect density is indicative of effective lattice strain relaxation facilitated by the substitutional incorporation of Bi atoms, which modulate the local atomic environment and enhance structural coherence. Further increasing the Bi content to 6% resulted in a slight reduction in crystallite size (64 nm) and a marginal increase in dislocation density (0.24 × 10^15^ m^–2^). Compared to bulk InSe samples, the screen-printed inks exhibited larger crystallite sizes.^14^ This can be ascribed to the solvent-assisted grain growth during ink processing, where gradual solvent evaporation and low-temperature curing promote enhanced atomic diffusion and grain coalescence, unlike rapid sintering in bulk synthesis, which often traps residual defects.^27^ These findings underscore that an optimal dopant concentration exists, beyond which the benefits of defect reduction and grain refinement may diminish due to dopant saturation effects. The 4% Bidoped sample exhibits enhanced crystallinity and a minimized defect landscape, indicating improved structural order that can be beneficial for charge carrier transport in subsequent thermoelectric performance.

### 3.2 FESEM and EDS Studies of Screen-Printed Ink Films

Figure 3 presents the FESEM micrographs showing the surface morphology of screen-printed InSe and Bi/Te codoped InSe thermoelectric films. All images were captured at a 1 μm scale to analyze the microstructural evolution induced by codoping and the printing process. The pristine InSe sample exhibits a compact but irregular microstructure, composed of densely packed grains and clusters. This disordered arrangement can hinder the transport properties due to increased grain boundary scattering. Upon introducing Bi and Te dopants, significant morphological changes are observed. At moderate doping levels, the film exhibits a mixture of small and large flake-like structures. This transition is likely driven by lattice strain or mismatch caused by dopant incorporation, which promotes enhanced phonon scattering and thus lowers lattice thermal conductivity. With increasing dopant concentration, the microstructure evolves into a more compact and uniform form. The grains appear better aligned, flake-like, and well-defined, indicating dopant-assisted grain growth and reorganization. This improved arrangement enhances interfacial contact and reduces porosity, both of which facilitate efficient charge transport, while maintaining sufficient phonon scattering at grain boundaries, an essential balance for optimizing thermoelectric performance. Notably, the FESEM images reveal a distinct layered morphology across all samples, more prominent at higher doping concentrations. This morphology reflects the intrinsic layered crystal structure of InSe,^28^ where stacked nanosheets are retained even after the screen-printing process. Such stratified architecture supports anisotropic transport behavior by enhancing in-plane electrical conductivity while increasing phonon scattering at interlayer interfaces, thereby reducing thermal conductivity. Additionally, the organic binder used in the ink formulation plays a vital role in shaping the final microstructure. It aids in particle dispersion and rearrangement during drying and annealing, leading to improved grain connectivity, reduced porosity, and strong film adhesion to the substrate. These features collectively contribute to enhanced electrical performance and mechanical stability in flexible devices.^29^ In summary, the microstructural transition from a disordered, agglomerated morphology to a more compact, layered, and uniform architecture with Bi/Te codoping underscores the combined effectiveness of the doping strategy and the screen-printing technique in optimizing the structural and functional properties of InSe-based thermoelectric films.

**Figure 3:**
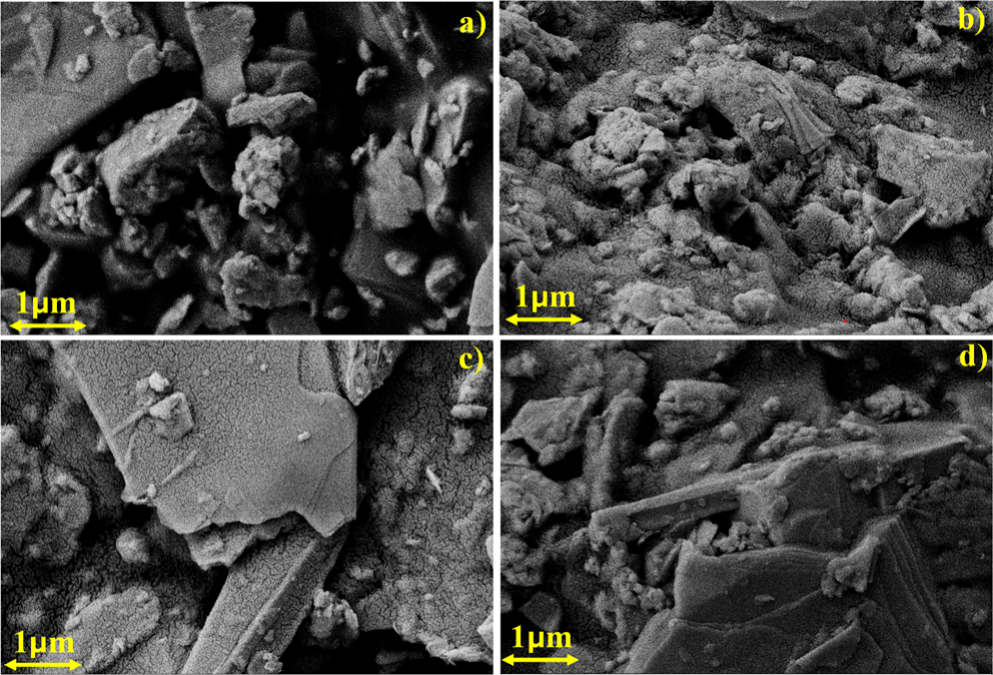
FESEM images of (a) InSe, (b) In_0.98_Bi_0.02_Se_0.97_Te_0.03_, (c) In_0.96_Bi_0.04_Se_0.97_Te_0.03_, (d) In_0.94_Bi_0.06_Se_0.97_Te_0.03_ ink film samples.

Figure 4 presents the energy dispersive X-ray spectroscopy (EDS) spectra and the corresponding atomic percentages of elements detected in pristine and Bi/Te codoped InSe ink films. The elemental composition confirms the successful incorporation of Bi and Te into the InSe lattice, providing insight into compositional variations across different doping levels. Each spectrum was acquired from the designated rectangular regions (highlighted in pink) shown in the inset FESEM images, ensuring localized analysis of representative microstructural areas. For the pristine InSe sample (Figure 4a), the EDS spectrum reveals the presence of only indium (In), selenium (Se), and carbon (C). The carbon signal arises from the organic binder used in the screen-printable ink formulation and is not associated with the intrinsic material composition. The nearly stoichiometric ratio of In and Se indicates high chemical purity and phase integrity of the pristine film. In contrast, the EDS spectra of the Bi/Te codoped samples (Figure 4b–d) clearly show the presence of Bi and Te in addition to In and Se, confirming successful elemental substitution. The consistent total atomic percentages across all samples suggest uniform doping without the introduction of unintended elements. Overall, the EDS analysis strongly validates the structural integrity and controlled elemental tuning of the screen-printed InSe-based thermoelectric films, thereby supporting the effectiveness of the adopted synthesis strategy in developing high-performance flexible thermoelectric generators.

**Figure 4:**
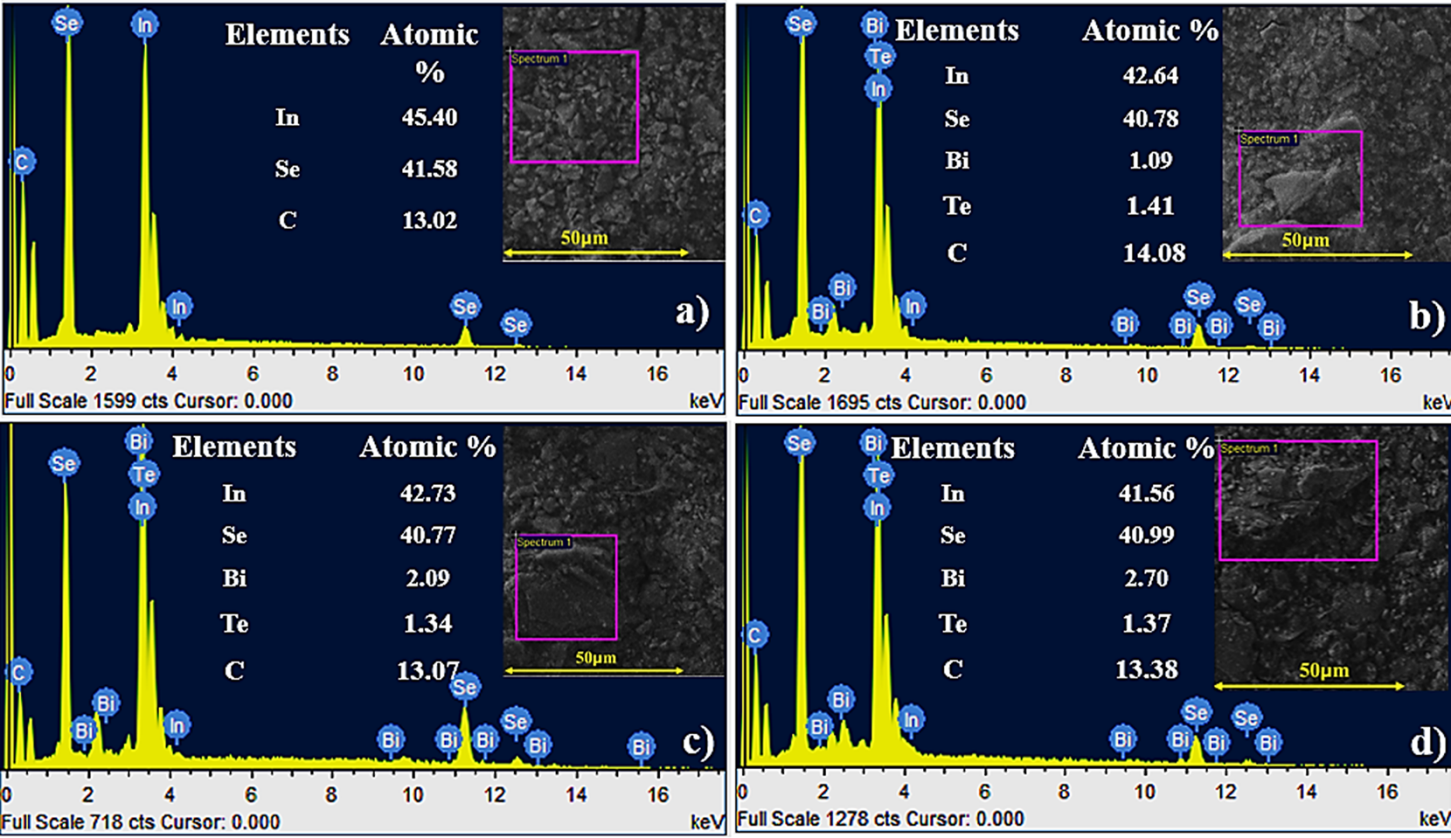
EDS mapping of (a) InSe, (b) In_0.98_Bi_0.02_Se_0.97_Te_0.03_, (c) In_0.96_Bi_0.04_Se_0.97_Te_0.03_, d) In_0.94_Bi_0.06_Se_0.97_Te_0.03_ ink films with spectrum collection location.

Figure 5a illustrates the measured thickness of screen-printed InSe and Bi/Te codoped InSe ink films. The results exhibit excellent uniformity across multiple sample regions, with thickness variation restricted to within 2.0%. This minimal variability highlights the precision and reproducibility of the printing process, which is critical for ensuring consistent thermoelectric performance. Thickness measurements were conducted using a high-accuracy Mitutoyo 547–301 digimatic thickness gauge. As demonstrated in our previous study,^18^ this contact-based measurement approach closely agrees with values obtained from cross-sectional FESEM imaging, thereby confirming its reliability. Furthermore, Figure 5b presents the cross-sectional FESEM image of the screen-printed In_0.96_Bi_0.04_Se_0.97_Te_0.03_ film, clearly confirming the uniform thickness and strong interfacial adhesion between the thermoelectric layer and the flexible substrate.

**Figure 5:**
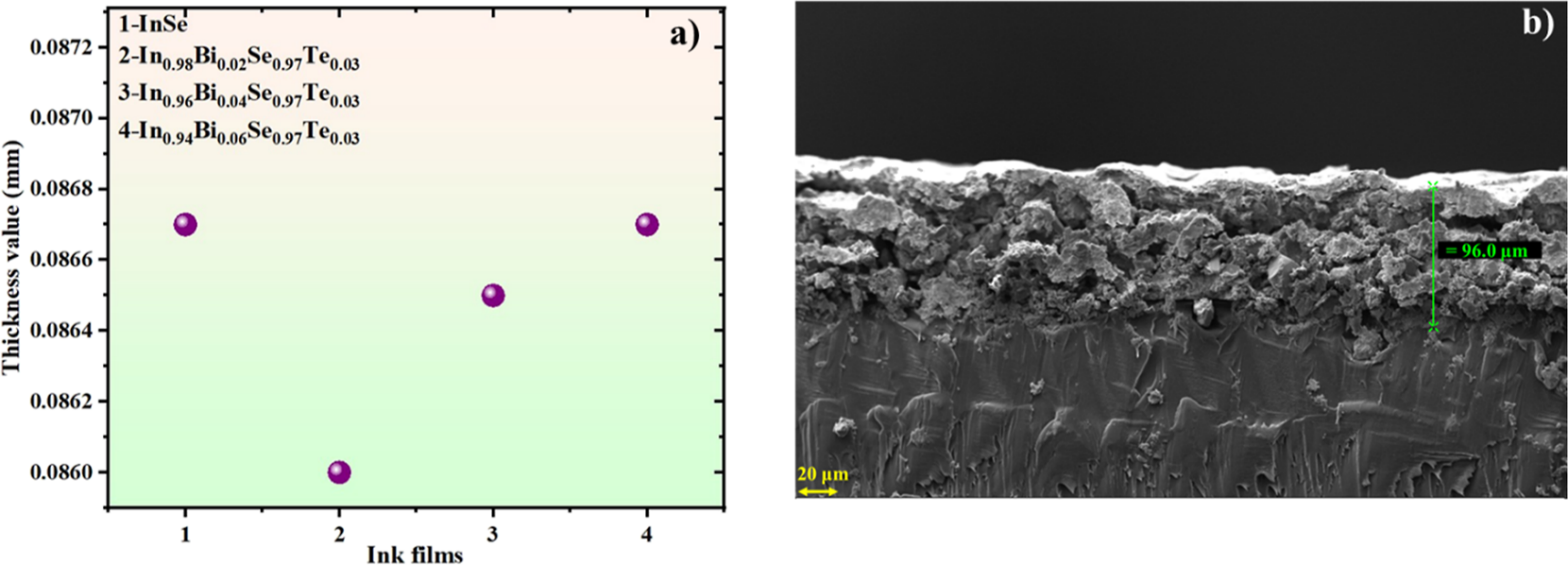
(a) Thickness values of InSe and Bi/Te codoped InSe ink films, (b) cross-sectional FESEM image of In_0.96_Bi_0.04_Se_0.97_Te_0.03_ film.

### 3.3 Electrical Transport Behavior via Hall Effect Analysis

Figure 6a displays the carrier transport properties of screen-printed In_1–*x*_Bi_*x*_Se_0.97_Te_0.03_ (*x* = 0, 0.02, 0.04, 0.06) samples, analyzed via Hall measurements at 300 K to evaluate the effect of Bi/Te codoping on electron concentration and mobility. All samples exhibit a negative Hall coefficient, confirming their n-type semiconducting nature, where electrons act as the majority carriers.^30^ This consistent n-type behavior across all compositions is attributed to the substitution of trivalent Bi^3+^ for In^3+^, introducing additional free electrons into the conduction band. The pristine InSe sample shows the lowest carrier concentration (1.03 × 10^17^ cm^–3^), which increases with doping, reaching a maximum (3.82 × 10^17^ cm^–3^) at *x* = 0.04. This increase reflects effective charge carrier generation via Bi-substitution.^12^ However, at higher doping (*x* = 0.06), the carrier concentration declines, possibly due to compensation effects such as defect complex formation or increased carrier trapping, which reduce the number of free carriers. Correspondingly, the mobility (μ) trend exhibits an initial decrease from pristine to *x* = 0.02 and 0.04, followed by an anomalous increase at *x* = 0.06. Notably, the crystallite size, as determined by XRD analysis, increases with doping and reaches a maximum at *x* = 0.04, indicating improved crystallinity and reduced grain boundary density. In general, larger crystallites are expected to enhance carrier mobility by minimizing boundary scattering. However, in the *x* = 0.04 sample, despite having the largest crystallites, the mobility is relatively low. This anomaly is attributed to the significantly increased carrier concentration (3.82 × 10^17^ cm^–3^), which introduces enhanced carrier–carrier and impurity scattering that overshadow the benefits of improved crystallinity.^31^ Thus, the dominant scattering mechanism at this doping level shifts from grain boundary scattering to Coulombic interactions and impurity-induced disruptions. At *x* = 0.06, although the crystallite size slightly decreases, a concurrent reduction in carrier concentration lessens the extent of carrier–carrier scattering, leading to a partial recovery in mobility. This highlights the delicate balance between crystallite size, carrier concentration, defect formation, and scattering mechanisms, emphasizing the need to optimize dopant levels to achieve efficient charge transport in flexible thermoelectric films.

**Figure 6:**
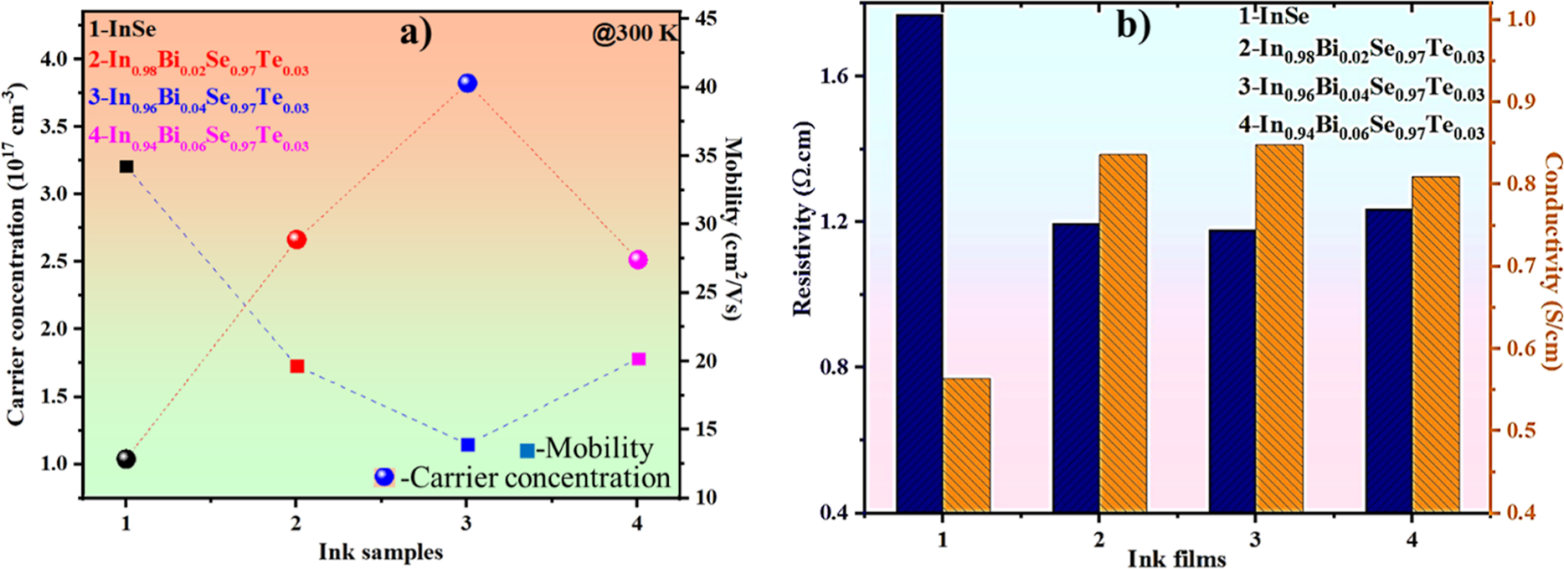
(a) Variation of carrier concentration and mobility, and (b) electrical resistivity and conductivity of the ink samples measured at 300 K.

The electrical resistivity and conductivity of the ink films, obtained via Hall measurement, exhibit a clear correlation with the dopant concentration. As shown in Figure 6b, the resistivity significantly decreases upon Bi and Te codoping, dropping from approximately 1.77 Ω cm in pristine InSe to around 1.17 Ω cm in the In_0.96_Bi_0.04_Se_0.97_Te_0.03_ sample. This is accompanied by a notable increase in electrical conductivity, reaching a maximum of ∼0.84 S/cm for the In_0.96_Bi_0.04_Se_0.97_Te_0.03_ sample. The improved conductivity arises from the enhanced carrier concentration induced by Bi doping; however, excessive doping (*x* = 0.06) shows a slight decline due to a decrease in carrier concentration. The reduced resistivity and elevated electrical conductivity contribute directly to enhanced thermoelectric performance by boosting the Seebeck coefficient and power output of the FTEG.

### 3.4 Analysis of Electrical Resistance, Seebeck Coefficient, and Power Output

Figure 7a illustrates the variation in internal electrical resistance as a function of temperature gradient (Δ*T*) for FTEGs fabricated from pristine InSe and Bi/Te codoped InSe inks. All devices exhibit a monotonic decrease in internal resistance with increasing Δ*T*, a characteristic behavior of thermally activated carrier transport typically observed in semiconductors.^32^ The pristine InSe FTEG shows the highest resistance across the entire Δ*T* range, primarily due to its low intrinsic carrier concentration and limited charge transport pathways. Upon Bi doping, a significant reduction in resistance is observed, from approximately 27.5 M Ω for pristine InSe to about 16 M Ω for In_0.96_Bi_0.04_Se_0.97_Te_0.03_ at Δ*T* = 116 °C, indicating a notable improvement in electrical conductivity. This enhancement is attributed to increased carrier concentration and improved crystallinity, as supported by Hall measurement and XRD analysis. However, further increasing the Bi doping to 6% results in a slight rise in internal resistance. This reversal is likely due to doping-induced lattice strain and the formation of point defects, as evidenced by XRD data. Correspondingly, Hall measurement reveals a reduction in carrier concentration at higher doping levels. These findings collectively indicate that 4% Bi doping offers the optimal balance, enhancing electrical performance without introducing excessive structural disorder that could impede charge transport.

**Figure 7:**
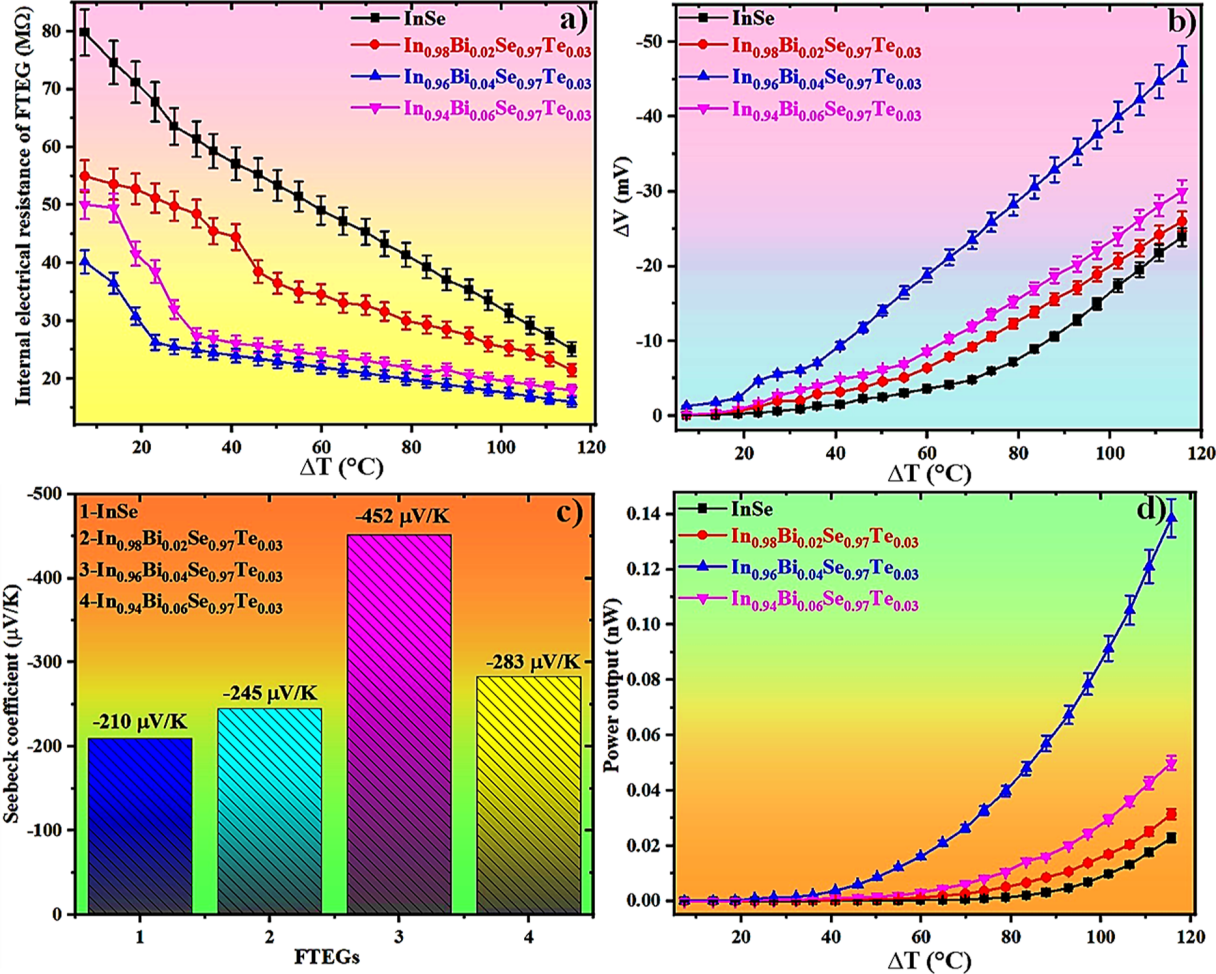
Trends in (a) electrical resistance, (b) Δ*V* as a function of Δ*T*, (c) Seebeck coefficient, and (d) power factor for the fabricated FTEGs.

Figure 7b displays the variation of internal voltage (Δ*V*) with temperature gradient (Δ*T*) for the fabricated FTEGs based on pristine and Bi/Te codoped InSe inks. All devices exhibit a consistent increase in Δ*V* with rising Δ*T*, which is characteristic of thermoelectric behavior driven by the Seebeck effect.^33^ The pristine InSe device generates the lowest voltage output across the entire temperature range, reflecting its low intrinsic Seebeck coefficient and limited carrier transport capability. Upon Bi doping, a marked enhancement in voltage output is observed. The In_0.96_Bi_0.04_Se_0.97_Te_0.03_ device exhibits the highest Δ*V*, reaching approximately 47 mV at Δ*T* = 116 °C, which is nearly twice that of the pristine InSe (24 mV) under the same thermal gradient. This improvement can be attributed to an optimized carrier concentration and enhanced electronic structure, which promote more efficient thermoelectric conversion. Interestingly, increasing the Bi content to 6% results in a moderate reduction in voltage output compared to the 4% doped device, which is due to increased scattering from excess dopant-induced defects and reduced carrier mobility.^31^ Figure 7c presents the Seebeck coefficient extracted from the linear fit of Δ*V* vs Δ*T* curves.^18^ The pristine InSe FTEG exhibits the lowest Seebeck coefficient of −210 μV/K, due to its limited carrier concentration and relatively poor band alignment. Upon Bi/Te codoping, a progressive improvement is observed, with the highest Seebeck coefficient of −452 μV/K recorded for the In_0.96_Bi_0.04_Se_0.97_Te_0.03_, more than double that of the InSe FTEG. This significant increase is attributed to enhanced carrier energy filtering and band structure modulation induced by Bi incorporation and Se-assisted vacancy formation.^34^ Notably, a further increase in Bi content to 6% slightly reduces the Seebeck coefficient to −283 μV/K, likely due to excessive lattice distortion and defect scattering, which hinder energy-dependent carrier transport.

These observations align with Hall and structural analyses, which reveal an optimal balance between carrier concentration and mobility at a 4% Bi doping level. The deviation from the ideal Pisarenko trend in pristine InSe, characterized by a low Seebeck coefficient despite low carrier density, is attributed to high defect density and ineffective band alignment.^34^ Co-doping with Bi and Te rectifies these limitations by tailoring the band structure and reducing scattering centers, thereby improving thermoelectric efficiency. Overall, these results confirm that moderate Bi/Te codoping (particularly at 4%) is optimal for achieving enhanced Δ*V* and Seebeck response in InSe-based FTEGs.

The power output (P) of the fabricated FTEGs was calculated using eq 4 ^17^

4 $$P=\frac{V^{2}}{R}$$

where *V* represents the voltage developed across the device (in volts), and *R* is the internal electrical resistance of the device (in ohms).

Figure 7d depicts the variation in power output as a function of temperature gradient (Δ*T*) for the fabricated InSe-based FTEGs. As Δ*T* increases, a general enhancement in power output is observed for all the devices. Notably, Bi/Te codoping significantly improves performance compared to pristine InSe, consistent with earlier trends in Δ*V* and Seebeck coefficient. The pristine InSe device yields the lowest power output of about 0.023 nW at Δ*T* = 116 °C. With the introduction of Bi, power output progressively increases, indicating improved thermoelectric performance. The In_0.96_Bi_0.04_Se_0.97_Te_0.03_ FTEG demonstrates the highest power output of approximately 0.14 nW, outperforming both the lower (2%) and higher (6%) Bidoped FTEGs. This optimal behavior is attributed to the synergistic effects of moderate Bi/Te codoping. Additionally, the observed reduction in internal resistance further contributes to improved power generation. Interestingly, the 6% Bidoped FTEG exhibits a decline in power output (∼0.05 nW), despite its higher Seebeck coefficient, likely due to increased carrier scattering from excess defects or dopant-induced localization effects.^35^ These observations underscore the crucial role of doping concentration in balancing electrical conductivity and thermopower to achieve optimal thermoelectric performance. The power output trend strongly correlates with the Δ*V* and internal resistance data, confirming the superior thermoelectric response of the In_0.96_Bi_0.04_Se_0.97_Te_0.03_ device. Table 2 showcases a comparative assessment of the Seebeck coefficient and power output of the fabricated In_0.96_Bi_0.04_Se_0.97_Te_0.03_ FTEG against previously reported values in the literature.

**Table 2:** Power Output Performance: Present Work Vs Reported Thermoelectric Materials

material	fabrication route	substrate	number of leg couples	Seebeck coefficient (μV/K)	power output (nW)	Δ*T* (K)	refs
Sb_2_Te_3_/Bi_2_Te_3_	screen printing	Kapton	4	282	195	20	^36^
PEDOT/PSS/Ag	inkjet printing	polyimide	8		0.24 × 10^–3^	5	^37^
PEDOT/PSS	solution coating	polyester fabric	5	18.5	12.29	75	^38^
CuI/GZO	Thermal evaporation	Kaoton CS	17		10.83	293	^39^
MWCNT-NH_2_/NiO	screen printing	PET film	15	55.27	1.44	100	^23^
Ag_2_Se/PVDF	physicalmixing + cold pressing	PI	5	95.9	5	30	^40^
Graphene	printing	paper	5	–21.5	1.7	60	^41^
AuCl_3_-doped P3HT	spin coating	flip film	20	163	1.9	10	^42^
PEDOT-Tos/carbon	inkjet printing	silicon	54	–48.0	1.13	308	^43^
Cu_2_Se	magneton sputtering + thermal evaporation	Kapton	10	55	3.3	38	^44^
CuI	sputtering	PET	1		8.2	10.8	^45^
InGaZnO	RF magnetic sputtering	PEN	2		0.12	53	^46^
Ni–Ag	thermal evaporation	silica fiber	7		2	6.6	^47^
Cu_2_S/PEDOT/PSS	vacuum filtration	PI	4	38.23	23	30	^48^
SnSe/PEDOT/PSS/MWCNT/DMSO	vacuum filtration	PI	5	22.3	14.7	39	^49^
In_0.96_Bi_0.04_Se_0.97_Te_0.03_	screen printing	PET film	8	–452	0.14	116	^a^

a Present work.

### 3.5 Investigation of Transient Thermal Transport Properties

The electronic thermal conductivity (κ_e_) in the FTEGs was estimated using the Wiedemann–Franz law: κ_e_ = *L*σ*T*,^50^ where *L* is the Lorentz number (2.45 × 10^–8^ W Ω K^–2^), σ is the electrical conductivity, and *T* is the absolute temperature. As illustrated in Figure 8, κ_e_ exhibits an increasing trend with temperature for all devices, which is attributed to enhanced carrier excitation at elevated temperatures. The undoped InSe FTEG exhibits the lowest κ_e_ values throughout the temperature range, indicating limited carrier concentration. With increasing Bi-substitution, a noticeable improvement in κ_e_ is observed. Specifically, In_0.96_Bi_0.04_Se_0.97_Te_0.03_ shows the highest κ_e_ among all devices, reaching approximately 0.004 W/mK at 115 °C, followed closely by In_0.94_Bi_0.06_Se_0.97_Te_0.03_. This enhancement reflects improved electrical transport properties due to optimal doping, which introduces additional charge carriers while maintaining structural integrity. However, beyond a certain doping level, such as in In_0.94_Bi_0.06_Se_0.97_Te_0.03_, the rate of increase in κ_e_ becomes less significant, likely due to carrier scattering or saturation effects. These trends highlight the critical role of Bi-substitution in tuning the electronic contribution to thermal conductivity, which is essential for optimizing thermoelectric performance.

**Figure 8:**
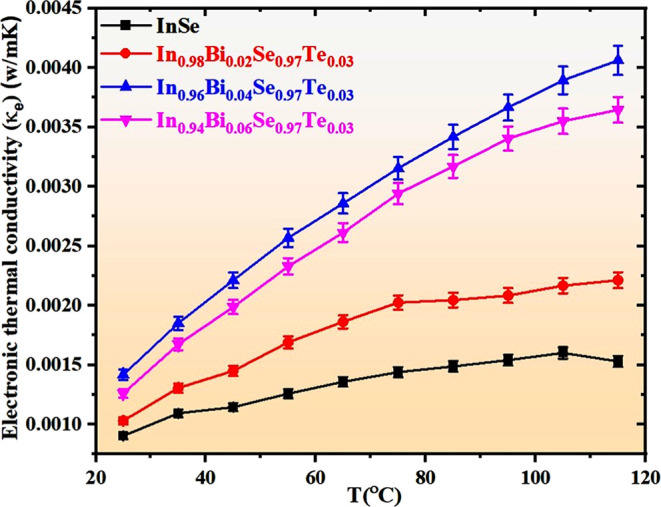
Plot of transient thermal conductivity (κ_e_) for the fabricated FTEGs.

While this analysis focuses on κ_e_, the total thermal conductivity (κ_tot_ = κ_e_ + κ_lat_) is expected to remain low due to enhanced phonon scattering from structural defects.^51^ The combination of high κ_e_ and suppressed κ_lat_ is key to boosting thermoelectric performance in flexible devices. Future work will directly assess κ_lat_ to better understand overall thermal transport.

### 3.6 Flexibility Assessment of FTEGs

The mechanical reliability and functional endurance of the screen-printed FTEGs were systematically evaluated under various static and dynamic mechanical deformation conditions, as illustrated in Figure 9a,b. To assess the electromechanical coupling behavior, the devices were subjected to controlled bending at static angles (0°, 30°, 60°, 90°, and 120°), during which the electrical resistance exhibited a slight variation of about ∼5%. This minimal resistance variation under mechanical flexure indicates that the strain-induced alterations in the percolation pathways of the conductive network are self-compensated by the inherent compliance of the printed thermoelectric films, preventing crack initiation or disruption of charge transport pathways.^52^

**Figure 9:**
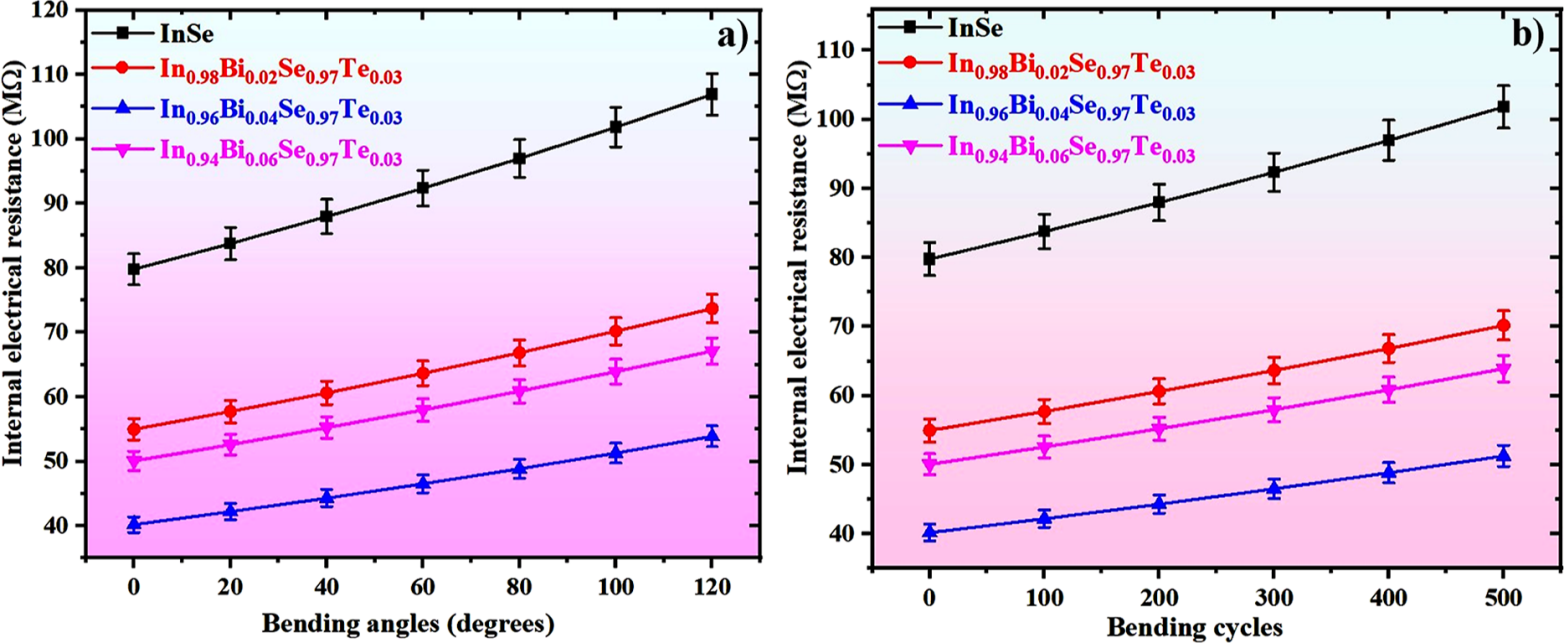
(a) Variation of internal resistance with bending angle, and (b) change in internal resistance as a function of bending cycles for the fabricated FTEGs.

Additionally, cyclic mechanical durability was tested over 500 repeated bending cycles, mimicking operational conditions typical of wearable and flexible electronics. The devices demonstrated remarkable mechanical resilience, with the electrical resistance showing less than a 5% variation throughout the cyclic loading. This indicates excellent fatigue resistance and microstructural stability, surpassing the degradation trends commonly observed in rigid or brittle thermoelectric materials reported in prior studies.^53^ The durability can be attributed to the synergistic role of the polymeric binder system and the solvent-mediated ink formulation, which promotes mechanical cushioning and mitigates stress localization during cyclic flexure.

Microstructurally, the dense yet flexible matrix of the screen-printed films distributes stress evenly, minimizing the risk of cracking or delamination. Moreover, the ink formulation and curing process ensure robust adhesion with the flexible PET substrate, ensuring a stable and long-lasting connection between the electrode and thermoelement, which is crucial for device reliability. These electromechanical stability results are crucial for practical deployment, especially in energy harvesting applications integrated with soft robotics, biomedical sensors, and next-generation wearable platforms, where devices must endure frequent dynamic mechanical stimuli without compromising electrical integrity. The negligible resistance variation observed postcycling confirms that the printed FTEGs can reliably sustain charge transport under repetitive mechanical stresses, thereby ensuring consistent thermoelectric output over prolonged operational periods.

## 4 Conclusions

In this work, Bi and Te-*co*-doped InSe was first synthesized using a conventional solid-state reaction method, ensuring phase purity and controlled stoichiometry. The doped powders were then used to fabricate flexible thermoelectric generators (FTEGs) via a low-cost, scalable screen-printing process under ambient conditions. The codoping strategy significantly improved the thermoelectric properties by tuning carrier concentration, enhancing the Seebeck coefficient, and optimizing electrical conductivity. The highest power output was achieved for the In_0_._96_Bi_0_._04_Se_0_._97_Te_0_._03_ device, delivering a Seebeck coefficient of −452 μV/K, an open-circuit voltage of 47 mV, and a power output of approximately 0.14 nW at a temperature difference of 116 °C, with an internal resistance of ∼16 MΩ.

In addition to its promising thermoelectric performance, the fabricated FTEG demonstrated excellent mechanical flexibility and durability, retaining over 95% of its original performance after 500 bending cycles and under different bending angles, underscoring the effectiveness of the material and fabrication strategy. This study showcases the potential of Bi–Te codoped InSe-based FTEGs made via a simple, low-cost screen-printing method as an effective route for developing flexible, high-performance thermoelectric devices suitable for energy harvesting applications.

Future work will focus on optimizing dopant concentration, enhancing long-term stability, mechanical durability, and integrating *p*–*n* pairs into flexible thermoelectric modules for improved device efficiency. These efforts will enable the practical implementation of the fabricated devices in smart textiles, biomedical sensors, and self-powered IoT systems.

## Data Availability

The data sets generated and/or analyzed during the current study are available from the corresponding author upon reasonable request.
